# Supplementary material for: Are carbon emissions trading and green financial instruments synergistic? -Comprehensive quantitative research based on content analysis
Source: PLoS One. 2024 Mar 7;19(3):e0298601. doi: 10.1371/journal.pone.0298601 (PMC10919601; doi:10.1371/journal.pone.0298601)
Supplement: S1 Data — (ZIP) [file pone.0298601.s001.zip › Data/地级市Z_F工作报告.zip!/╣≤╓▌/╫±╥σ╩╨/└┤╫╘╣1⁄2╓┌║┼í╛┬φ┐╦╩2╛▌═°í┐_2018.docx]

各位代表：

　　现在，我代表市人民政府向大会作工作报告，请予审议，并请各位政协委员和其他列席人员提出意见。

　　2017年工作回顾

　　刚刚过去的2017年，党的十九大胜利召开，描绘了决胜全面建成小康社会、夺取新时代中国特色社会主义伟大胜利的宏伟蓝图。一年来，我们紧密团结在以习近平同志为核心的党中央周围，在省委、省政府和市委的坚强领导下，深入学习贯彻党的十九大精神和习近平总书记治国理政新理念新思想新战略，统筹推进“五位一体”总体布局，协调推进“四个全面”战略布局，坚持稳中求进工作总基调，深入践行新发展理念，坚守发展和生态两条底线，坚定信心、真抓实干，顺利完成了年初确定的主要目标任务。

　　——经济持续稳定增长。初步统计：完成地区生产总值2748.6亿元，增长12.1%；规模工业增加值1102.9亿元，增长11.7%；500万元以上固定资产投资2523.6亿元，增长22%；社会消费品零售总额811.7亿元，增长12.2%；财政总收入和一般公共预算收入586.3亿元、216.4亿元，分别增长22.1%、17.9%；城乡居民人均可支配收入29617元、11130元，分别增长9.3%、10.1%；金融机构存贷款余额4661.2亿元、2792.3亿元，分别增长8.3%、23.3%；居民消费价格指数控制在省控目标内；全面小康实现程度92.5%。

　　——脱贫攻坚成效明显。减少农村贫困人口15.33万人，318个贫困村按国标脱贫退出，贫困发生率由5.76%下降到3.5%，贫困县农村居民人均可支配收入突破9000元。赤水市在全省率先通过国家脱贫评估验收，“赤水经验”得到中央领导肯定。

　　——文明创建开创新篇。社会主义核心价值观深入人心，全国文明城市创建成功，9个村镇列入全国文明村镇，5家单位获评全国文明单位，3所中小学校成为首批全国文明校园，涌现出黄大发等时代楷模和全国道德模范，遵义好声音、好故事不断传颂。

　　——发展动能蓄势待发。供给侧结构性改革深入推进，遵义综合保税区正式获批，遵义院士工作中心正式成立，4个院士、专家团队汇聚名城，遵义国家高新技术产业开发区通过专家组评估验收，国家级科技创新企业孵化器实现零突破。

　　——通江达海全面提速。遵义新舟机场年旅客吞吐量达到156.1万人次，茅台机场正式通航，贵遵复线、道南高速建成通车，渝贵铁路通车在即，“铁公机”联动的高速交通体系正在形成，全面进入了“双机场”和高铁新时代。

　　——社会治理保持领先。连续第4次荣获“长安杯”、第6次得到全国社会治安综合治理优秀表彰、第4年进入中国30个最安全城市、第8次蝉联“全国双拥模范城市”称号，人民群众安全感不断增强，安全测评位居西部城市前列。

　　——遗留问题大量化解。重拳出击整治问题楼盘，维护了群众的切身利益。严厉打击非法集资，处置非法集资案件26起。铁腕整治环保遗留问题，31个突出问题得到有效整改。大力化解信访积案，市、县两级领导干部累计接访4210人次，解决了一批长期应解决而未能解决的问题。

　　——醉美遵义好戏连台。第三次全国改善农村人居环境工作会议在湄潭县成功召开。遵义绿色农产品获北京、上海等市场青睐。茶博会、辣博会、旅发会、酒旅会等展会活动精彩纷呈。国际半程马拉松、国际户外运动挑战赛等体育赛事亮点频闪。成功举办道真自治县、务川自治县成立30周年纪念活动，彰显了民族团结、社会和谐的精神面貌。遵义撤地设市20周年系列活动进一步提振了全市人民的精气神。

　　从这些发展的变化和取得的成绩可以看到，一年来我们集中精力抓大事、解难事、干实事，发展环境不断优化，经济韧性不断增强，群众获得感不断提升。这一步一个艰辛的脚印，标志着我们在决胜全面建成小康社会的进程中迈出了坚实步伐，正以更加从容自信的姿态阔步迈进中国特色社会主义新时代。

　　一年来，我们主要抓了以下工作：

　　一、全面精准聚焦深度贫困，大扶贫战略行动成果丰硕。坚持以脱贫攻坚统揽经济社会发展全局，紧扣五个一批⑴、六个精准⑵，扎实推进春季攻势、夏季大比武和秋季攻势，主攻四场硬仗⑶，打好八大战役⑷。争取省级扶贫产业基金102.8亿元，贫困户基本实现“一长两短”产业全覆盖。建立贫困户利益联结机制，累计带动35.6万贫困人口就业。建成“组组通”公路4316公里。实施易地扶贫搬迁10.2万人，户均就业1.5人，旧房拆除率达91%。改造农村危房5.7万户。解决了56万农村群众饮水安全问题。落实教育扶贫资金12亿元，资助学生93万人次、为65万农村学生提供营养补助，培训农村贫困劳动力1.7万人次。落实建档立卡贫困人口“四重医疗保障”⑸，政策范围内报销比例93%以上。城乡低保分别提标10%、15.3%。上海对口帮扶成果丰硕。“城市解困·和弦行动”成效初显。

　　二、着力加快数字经济发展，大数据战略行动扎实推进。全面推动大数据“聚通用”，积极引进大数据企业，培育智能终端规模企业，大力发展电子信息制造业，打造智能终端全产业链，大数据产业规模总量达到808亿元、增长27.2%。推进大数据“触角”向三次产业和产业链各个环节延伸，大数据与实体经济融合发展水平位居全省前列。完善电商服务体系，培育电商骨干企业28家，电商交易额增长35%。劳动用工监管综合服务云等大数据云平台建成投用。加强信息基础设施建设，新增光缆线路1.6万公里，95%以上的建制村通宽带。推进大数据便民服务，乡镇（街道）网上办事大厅服务站点实现全覆盖，村（居）便民服务站覆盖率100%。

　　三、加大生态环境保护和改善力度，大生态战略行动成效明显。牢固树立生态优先、绿色发展理念，书写绿水青山就是金山银山大文章，“治污治水·洁净家园”五年攻坚行动扎实推进。完成营造林128.4万亩，森林覆盖率59%。湿地公园、山体公园、乡镇森林公（游）园、生态体育公园建设积极推进。全面实施“河长制”，湘江系统整治工程全面启动，乌江流域56座乡镇污水处理厂全面开工，治理中小河流14条、水土流失70余平方公里。用水总量、用水效率、限制纳污“三条红线”低于省控目标，县城以上集中式饮用水源水质达标率100%。工业固废综合利用率60%以上，城乡生活垃圾无害化处理率89.4%，医疗废弃物安全处置率100%。中心城区环境空气质量优良率94.2%。

　　四、大力发展实体经济，稳增长抗风险能力不断增强。深入实施“千企引进”“千企改造”工程，引进亿元以上项目367个，新入库规模工业企业99户，规模工业企业利润占全省比重55%。加大市场主体培育力度，新设立市场主体11.5万户，新增注册资本1267亿元，分别增长63.4%、25.6%，民营经济比重提高到56%。精心组织开展各类招商引资活动，引进500强企业17家，招商到位资金增长15.8%。加大品牌创建力度，建成国家级出口食品农产品质量安全示范区1个，新增地理标志保护产品7个。创新政府性债务管控机制，政府性债务总体可控，经济发展韧性增强。

　　五、全力加快产业转型升级，发展质量和效益不断提升。大力推进产业融合发展，调整优化产业结构，三次产业结构比例为14.6:45.2:40.2。围绕建设“三区三中心”⑹目标，研究制定了加快第三产业发展的“1+9”政策和制度体系，储备了975个第三产业重点项目，三产增加值增长14.3%。全面推进国家全域旅游示范区建设，新增4A级景区5个，“厕所革命”连续3年获得国家表彰，接待游客人次和旅游综合收入分别增长39.5%、44.2%，赤水河谷旅游度假区列入国家旅游度假区。大力推动白酒、电力、煤炭、装备制造、铝及铝加工等产业转型升级，“遵义十大名酒”⑺精彩亮相。完成工业投资700亿元，新建标准厂房350万平方米，130个重点工业项目建成投产。加快农业产业结构调整，促进绿色农产品“泉涌”发展，遵义红、湄潭翠芽、虾子辣椒等品牌影响力进一步提升；累计流转土地220万亩，调减低效籽玉米65万亩，粮经比为38:62；新增市级产业化经营龙头企业67家，累计认证“三品一标”⑻产地1218个740万亩、产品635个；创建国家标准化示范区3个；农产品加工业产值达到410亿元，一产增加值增长6.8%。

　　六、狠抓城市规划和基础设施建设，城乡协调发展支撑力增强。确立了“黔川渝结合部中心城市”的战略定位、“山水相望、宜居宜业宜游生态城市”的发展定位、“遵道行义·醉美遵义”的形象定位，城镇化思路更加清晰，进程不断加快，常住人口城镇化率达49%以上。新一轮城市总规、中心城区综合交通、重点区域控制性详规和城市设计等规划编制工作全面推进。实施10.1万户城镇棚户区改造，推进21个城市道路项目建设，改造中心城区小微循环道路33公里、社区排污管网65公里，新增公共停车位1.4万个。统筹推进特色小城镇和美丽乡村建设，鸭溪镇、永兴镇入选“中国特色小镇”。全面加大基础设施建设投入，改造国省道300公里、县乡道100公里，硬化撤并建制村道路5003公里，实现村村通油（砼）路和客运。治理病险水库11座，9座在建水库下闸蓄水，新开工中小型骨干水源工程10个。

　　七、扎实推进改革开放创新，经济发展活力竞相迸发。全面落实国家和省、市各项改革试点任务，研究推动31项重大改革措施，降低用电成本9亿元，减税降费139亿元；深入推进投融资体制改革，新增“新三板”挂牌企业4家，全省第2家金融资产管理公司落户遵义；强力推进国企改革，26家市属国有企业优化重组为“四大集团公司”⑼；全面深化农村集体产权制度改革，土地承包经营权确权颁证基本完成，187个村实施农村“三变”⑽改革试点；大市场、大科室、大轮岗、高中（中职）学校和公立医院去行政化、综合行政执法等改革全面推进。遵义新舟机场出入境3.2万人次；累计实现外贸进出口总额11.5亿美元、增长114%，新增外贸企业70家；实际利用外资3.9亿美元。遵义软件（大数据）产业技术创新战略联盟成立，国家低压电器产品质量监督检验中心（贵州）挂牌。主导编制了《黔北民居》省级地方标准，贵州钢绳集团牵头制定的行业标准实现了中国钢丝绳行业和贵州国际标准零的突破。新增市级科技创新平台11个、省市级科技创新人才团队11个、省级工程技术研究中心3个，高新技术企业总量突破100家，申请发明专利1200件，综合科技进步水平指数70.7%。

　　八、千方百计保障和改善民生，群众获得感进一步增强。大力促进教育优先发展，农村义务教育阶段学生、集中连片特困地区学前教育儿童营养改善计划实现全覆盖，自筹资金启动了非集中连片特困地区学前教育儿童营养改善计划，6个县域内义务教育均衡发展通过国家评估验收，建成公办幼儿园120所、“全面改薄”⑾项目100个，茅台学院、北京师范大学遵义附属学校建成招生，中国足球运动学院西南分院落户遵义。着力织牢民生保障网底，新增城镇就业14.4万人，转移农业劳动力12.9万人，城镇登记失业率控制在4.2%以内。医疗“五个全覆盖”⑿巩固提升，“百院大战”⒀持续推进，跨省异地就医实现直接联网结算。人口出生率14.4‰。市科技馆、青少年活动中心、“三线建设”⒁博物馆建成投用。奥体中心等省“十运会”项目建设和筹备工作全面推进。《遵义市志》出版发行。免费法律援助8111件。“三位一体”⒂立体化治安防控体系基本形成。生产安全事故和死亡人数连续17年实现“双降”。“十件民生实事”基本完成。同时，外事侨务、民族宗教、国防教育、人民防空、防灾减灾、档案管理、应急管理、妇女儿童、残疾人、地方志、红十字会等事业全面进步。

　　九、始终坚持从严从实治政，政府自身建设进一步强化。牢固树立“四个意识”，坚定“四个自信”，推进“两学一做”学习教育常态化制度化，党的十九大精神不断内化于心、外化于形，自觉在思想上政治上行动上同以习近平同志为核心的党中央保持高度一致。大力发扬“钉钉子”精神，推动各项决策部署项目化、数据化、目标化落实，深入推进脱贫挂帮、企业帮促，责任意识、担当意识不断增强。全面推进依法行政，强化法制审查、行政复议、法律顾问等工作，行政违法和行政不当行为得到及时纠正。大力提高行政效能，深化“放管服”⒃改革，市级行政许可事项平均办理时限压缩57.7%。认真贯彻《准则》和《条例》，严格执行市委十二项规定，持之以恒纠正“四风”。强化审计监督和民生监督，“三公”经费⒄缩减10%。自觉接受监督，认真办理人大代表建议、政协委员提案。密切联系群众，群众信访问题得到妥善化解。

　　各位代表，过去的一年，我们付出了艰辛和努力，也收获了成功和喜悦，一年的发展进一步启示我们，决战脱贫攻坚，决胜全面小康，是现阶段最根本的任务和最光荣的使命。为此，我们必须坚定不移时刻维护核心，全面增强“四个意识”，坚决维护习近平总书记作为全党的核心地位，自觉把思想和行动统一到党的十九大精神上来，时刻不忘用习近平新时代中国特色社会主义思想武装头脑、指导实践。必须守好发展和生态两条底线，坚持稳中求进工作总基调不动摇，以供给侧结构性改革为主线，全力打好风险防范、脱贫攻坚、污染治理三大攻坚战，努力在后发赶超中转型跨越，做到既要绿水青山又要金山银山，实现两条底线一起守、两个成果一起收。必须落实以人民为中心的发展思想，坚持把民之所望作为施政所向，尽力而为、量力而行，针对人民群众关心的突出问题精准施策、持续发力，加快补齐民生短板，增强群众获得感，不断满足人民群众日益增长的美好生活需要，实现经济发展与民生福祉互促共进。必须牢固树立遵义思维，不忘初心、牢记使命，面对省情、立足市情，以追求卓越的精神状态和舍我其谁的奋斗姿态，雷厉风行、久久为功，发扬长征精神和遵义会议精神，牢牢掌握后发赶超主动权，把遵义的新变化书写在黔北大地上。

　　各位代表，这些成绩的取得，得益于党中央、国务院的亲切关怀，得益于省委、省政府和市委的坚强领导，得益于市人大、市政协的监督支持，得益于全市干部群众的艰苦奋斗，得益于社会各界的鼎力相助，得益于上海等城市的对口帮扶和支持。在此，我谨代表市人民政府，向全市各族人民，向人大代表、政协委员，向各民主党派、工商联、人民团体、无党派人士，向驻遵人民解放军、武警部队官兵、公安干警，向所有关心支持遵义经济社会发展的各界人士，表示衷心的感谢！

　　在充分肯定成绩的同时，我们也清醒地看到，遵义面临的发展任务仍然艰巨繁重，问题和挑战不容忽视。主要是：发展不平衡不充分的矛盾突出，发展的质量和效益还需要提高；社会投资不旺，投资保持高速增长的压力较大；脱贫攻坚任务艰巨，产业脱贫底子薄弱；实体经济发展面临的困难较多，抗风险能力不强；新旧动能转换步伐较慢，发展后劲不足；现代服务业发展相对滞后，结构调整的任务仍然艰巨；各种体制机制还需进一步完善，防范化解和管控各类风险的能力还要不断提高；干部“本领恐慌”仍然存在，“四风”问题偶有露头；教育医疗、文化体育、生态环保等公共服务水平不高等。所有这些问题，都需要我们始终以问题为导向，着力研究问题，坚持破解难题，持之以恒发力，务求大力解决。

　　2018年工作的总体要求

　　2018年是贯彻党的十九大精神的开局之年，是改革开放40周年，是决胜全面建成小康社会、实施“十三五”规划承上启下的关键一年。做好今年的工作，意义十分重大。

　　今年工作的总体要求是：深入学习贯彻党的十九大精神，以习近平新时代中国特色社会主义思想为指导，坚持稳中求进工作总基调，坚持新发展理念，紧扣社会主要矛盾变化，按照高质量发展要求，统筹推进“五位一体”总体布局，协调推进“四个全面”战略布局，以深化供给侧结构性改革为主线，全面抓好稳增长、促改革、调结构、惠民生、防风险各项工作，大力推动质量变革、效率变革、动力变革，全力打好三大攻坚战⒅，深入实施三大战略行动⒆，守好发展和生态两条底线，促进经济社会持续健康发展，为2020年与全国同步建成全面小康社会奠定坚实基础。

　　推动高质量发展是新时代工作的根本要求，实现高质量发展是决战脱贫攻坚、决胜全面小康的根本出路，也是我市当前和今后一个时期发展的现实需要。遵义作为西部地区，特点是欠发达、欠开发、欠开放、欠账多，把握高质量发展的内在要求，必须正确处理好质量与速度、全国大局与遵义实际之间的关系，充分认识到发展是第一要务，高质量是目标、是方向，把发展的着力点转向“质”的提升，稳住“量”的增长。在质的提升上，坚持以供给侧结构性改革为主线，坚定不移发展实体经济，提升白酒、茶叶、绿色农产品和良好生态环境等有效供给水平，做足长板，不断满足人民群众个性化、多样化的需求。在量的增长上，坚持把扩大投资规模作为稳增长的主抓手，着力做大做强新型工业、做优做活服务业、做实做特现代农业，加大脱贫攻坚、产业发展、城乡基础设施、教育医疗文化等民生事业投资力度，补齐短板，努力让投资有回报、企业有利润、员工有收入、群众有就业，确保在较长时期内保持一个高于全国、高于西部、高于全省，更高质量、更有效率、更加公平、更可持续的增长速度。

　　三大攻坚战是不可割裂的有机统一体，打好三大攻坚战是决胜全面建成小康社会的根本保证。防范化解重大风险是推动高质量发展和稳中求进的基础。必须切实增强风险意识，提高风险防控能力，着力防范化解政府债务、国有企业债务、金融、安全生产、社会矛盾等重点领域风险，未雨绸缪，防患于未然，坚决打好防范化解重大风险攻坚战。脱贫攻坚是头等大事和第一民生工程，是输不起的攻坚战。必须聚焦再聚焦、精准再精准，始终坚持“一达标两不愁三保障”⒇脱贫标准，向深度贫困聚焦，向四场硬仗发力，以贫困不除愧对历史的使命感、群众不富寝食难安的责任感，坚决打赢脱贫攻坚战。良好的生态环境是遵义发展的最大优势，是我们坚守的底线。必须以对人民群众和子孙后代高度负责的态度，牢固树立绿水青山就是金山银山的理念，坚持醉美遵义、拒绝污染，推动生态优先、绿色发展，打好污染防治攻坚战，努力让天更蓝、地更洁、山更绿、水更清、气更净、生态环境更美好。

　　2018年工作的主要任务

　　按照稳中求进工作总基调和高质量发展总要求，今年的主要预期目标是：增比进位综合测评继续保持在全省第一方阵。确保地区生产总值增长12%以上，规模工业增加值增长11.5%，500万元以上固定资产投资增长21%以上，社会消费品零售总额增长12%，财政总收入、一般公共预算收入分别增长10%，城乡居民人均可支配收入分别增长9%、10%，金融机构存贷款余额分别增长10%、18%，全面小康实现程度达到95%，各县（市、区）人均地区生产总值达到同步小康创建年度目标。居民消费价格指数、节能减排降碳等指标控制在省调控目标内。

　　围绕总体要求和预期目标，我们必须抓好十个方面的工作：

　　一、全力打好脱贫攻坚战。强化精准扶贫、精准脱贫。进一步压实扣牢大扶贫责任链任务链，确保湄潭、习水、凤冈、桐梓脱贫摘帽，务川、道真达到国家第三方评估退出标准，111个以上贫困村出列。统筹抓好非贫困县贫困人口脱贫，减少农村贫困人口6万人以上。打好四场硬仗、八大战役。坚持强龙头、创品牌、带农户，用足用好扶贫产业子基金，大力推进“一县一业” “一村一品”，加快“一长两短”产业进村入户，建立完善产销对接机制，推动全产业链发展、全利益链联接，促进贫困群众增收致富。推进“四好农村路”（21）建设，新增“组组通”公路8000公里以上。新增易地扶贫搬迁2.4万人。全面落实教育扶贫政策，完成农村贫困劳动力全员培训2.4万人以上。全面落实“四重医疗保障”，确保应助尽助。坚持应保尽保、动态管理、应退则退，推动城乡低保标准分别提高6%、9%以上。抓实扶贫协作、社会帮扶。承接落实好上海市对口帮扶任务，推动在上海市建立稳定的绿色农产品销售中心和供应渠道，建立旅游联盟、劳务输出和产业合作伙伴。深化干部驻村、结对帮扶，扎实推进民营企业“千企帮千村”精准扶贫行动。持续抓好“城市解困·和弦行动”。着力办好新时代农民(市民)讲习所,提振群众脱贫致富信心,提高自我发展能力。统筹推进以县为单位同步小康创建，提升核心指标和基础性指标创建水平。

　　二、因地制宜实施乡村振兴战略。科学编制乡村振兴规划。始终把解决“三农”问题作为重中之重，围绕“产业兴旺、生态宜居、乡风文明、治理有效、生活富裕”总要求，因地制宜制定乡村振兴指导意见、编制战略规划。坚持以“四在农家·美丽乡村”小康行动升级版为总抓手，支持余庆、湄潭、凤冈、绥阳、赤水开展乡村振兴试点，全面推进村庄规划和农村人居环境整治。优化调整主导产业布局。围绕提高三个比重（22），大规模调整农村种养结构，推动旱地、基本农田种植经济作物，改变种植玉米的传统习惯。着力巩固烤烟产业，做大做强茶叶、蔬菜（辣椒、食用菌、方竹笋）、生态畜牧水产、中药材、干鲜果等五大主导产业，稳定茶园面积206万亩，种植辣椒225万亩、食用菌3亿棒，新造方竹97.2万亩、低产改造11.3万亩，实现畜牧渔业总产值200亿元，中药材种植面积180万亩，以县为主加快发展石斛、构树、优质稻、精品水果等区域特色产业。大力推进农产品精深加工，全力培育休闲农业、观光农业、设施农业等新业态，积极发展农村电商、快递物流、仓储、金融等新模式，创建县级以上农业园区170个，建成农旅一体化示范点100个。构建现代生产经营体系。全面推进良种化、机械化、科技化、信息化、标准化生产体系建设，农业机械化综合水平达39%，科技贡献率达55%，新增无公害产地认证100万亩、产品120个和绿色产品50个、有机产品40个、地理标志保护农产品2个，创建2个国家级农产品质量安全县。大力培育新型经营主体，建立以村为基本单位的农业农村发展组织体系和推进机制，全面推广“公司+合作社+农户”模式，新增土地流转100万亩，新增市级以上龙头企业70家、庄园经济经营主体30个、专业合作示范社100个，实现村社合一全覆盖。深化农业农村综合改革。积极稳妥推进农村集体产权制度和农村土地“三权分置”（23）改革，每个乡镇建立1个以上“三社融合”（24）发展合作社，打造农村“三变+N”改革发展升级版。实施村（社区）基层组织“减负固本行动”，推进自治、法治、德治相结合的乡村治理体系建设，全面提高农村基层治理水平。

　　三、聚焦聚力工业振兴。坚持两轮驱动（25）。深入推进“百千万”工程和“民营企业服务年”活动，持续开展双培育、双退出、降成本行动，帮助企业破解用能、用地、用工、融资、物流等难题。实施国有工业企业振兴计划，加快遵义钛业、贵州钢绳、天仪电器等企业“退城进园”。实施白酒产业振兴计划，支持茅台集团发展，加大“遵义十大名酒”营销力度，推进全国重要名酒产业集聚区建设。实施军民融合产业发展规划，加大民参军推进力度，全力打造国家级军民融合发展试验区。实施双千工程。实施“千企改造”项目100个，确保建成投产40个，新增省级企业技术中心2个以上。完成新一轮煤矿兼并重组，积极推进煤矿机械化智能化改造。加快推进铝加工项目建设，延长铝产业链。加大茶叶、食品、健康水等特色优势产业扶持力度，强化“专精特新”中小企业培育，打造茶叶加工集聚区。大力推进“千企引进”，扎实开展“产业大招商突破年”活动，着力引进一批技术含量高、成长性好、引领性强的国内外优强企业，招商引资到位资金增长14%以上。推动两化融合（26）。深入推进“遵义智造”行动，加快智能终端产业和新能源汽车产业集聚区建设，积极申报“中国制造2025”国家级示范区，高新技术产业增加值增长20%。加强“绿智园区”建设，完善园区基础及配套设施，新建标准厂房200万平方米以上，盘活用好现有园区标准厂房。强化园区主导产业培育和市场化运作，创建一批循环经济和清洁生产示范企业、示范园区。

　　四、加快第三产业发展。加快旅游康养集聚区建设。编制完成全域旅游发展规划、遵义桃花江健康旅游示范基地规划。大力实施“创A工程”，重点推进95个3A级景区创建，力争各县（市、区）建成1个以上4A级景区，力争赤水丹霞旅游区成功获批国家5A级景区，规划建设黔北渝南精品旅游线，促进旅游业“井喷式”增长，旅游接待人次和综合收入分别增长30%、32%以上。实施入境旅游市场开拓三年行动计划,推动设立境外旅游营销中心和航空旅游海外营销联盟，入境游人次增长20%。每个县（市、区）建成1个美食城（街区）。加快金融集聚区建设。全力打造金融商务区、金融小镇、金融街区，新增金融机构4家以上，金融增加值增长18%以上。实施企业上市“百千行动计划”（27），实现中小板挂牌企业1家，新增新三板挂牌企业10家。加快大数据服务集聚区建设。抢抓全省建设国家大数据综合试验区机遇，推动建设遵义智慧大数据资源（块数据）中心，开展政府大数据融合，促进数据“聚通用”，建成遵义“数据铁笼”（28）试点工程。培育和引进一批软件和信息技术服务企业，实现软件和信息服务业增长20%以上。构建多维度数据资源体系，推进大数据深度应用，助推实体经济转型升级，形成“大数据+”全域融合的发展格局。加快商贸物流中心建设。推进“一园区两中转四层级”（29）快递物流体系建设，建成黔北快递物流园区和11个县级快递物流分园。着力做大做强县域商圈，在每个县（市、区）建设1个农商旅综合体。推动电子商务增量提质，新建2个电商园区，电商交易额增长35%以上。新入库限上商贸企业100户。加大冷链物流建设力度，实现县级和主要产业基地冷库全覆盖。推动虾子辣椒城创建集鲜椒、干椒、辣椒制品和种子交易为一体的国家级专业批发市场。在中心城区规划建设1个集花鸟虫鱼、古玩字画等展示交易为一体的大型专业市场。加快会展中心建设。探索市场化、企业化机制，加大会展场馆建设力度，充分用好现有各类场馆资源，创新办好茶博会、辣博会、旅发大会等展会。加快文化中心建设。深入推进国家文化消费试点城市创建，建成市美术馆、大剧院、文化馆、公共服务中心和“三馆合一”项目（30），拍摄《伟大的转折》《花繁叶茂》等影视剧，打造“中国·长征园”“中国傩城”等一批文化产业示范园区。

　　五、持续深入推进新型城镇化。加强城市规划管控。加快完善城乡规划体系，完成《遵义市城市总体规划（2017—2035）》修编，推动各县（市、区）新一轮总规修编，推进中心城区总体规划、土地利用规划“两图合一”和控规全覆盖。加快城市规划信息平台建设，依法严厉打击“两违”（31）建设行为。加快中心城区与仁怀、湄潭、桐梓、绥阳同城化发展，积极推进绥阳、湄潭撤县设区和习水撤县设市。常住人口城镇化率提高到51%以上。完善城镇功能设施。强力推进中心城区路网项目建设，统筹抓好城市“双修”（32），积极推进海绵城市、地下综合管廊建设，新增中心城区停车位1.3万个，启动16个海绵型示范道路、17个示范公园、16个示范小区、17条生态景观示范河道建设，不断改善老城区人居环境，完善新区生产生活配套，加快建新城、疏老城步伐。实施“提质培优·美丽县城”计划，推动山水生态特色卫星城市群建设。坚持“三生融合”（33），抓好2个国家级、3个省级新型城镇化示范区建设，巩固提升56个列入省、市级示范小城镇，整体推动建设125个特色小城镇，每个县（市、区）重点打造2个以上特色示范小城镇。提升城市管理水平。不断总结文明城市创建经验，巩固提升全国文明城市创建成果，坚持标准不减、力度不降、管理更严，建立健全常态化、长效化机制，精细化推进城市综合管理。建立科学的房地产市场管理机制，积极推进新建商品房精装修销售，促进房地产市场平稳健康发展。

　　六、全面加大基础设施建设力度。加快水陆空交通基础设施建设。启动遵义新舟机场二期改扩建工程，加快赤水、正安、播州等通用航空机场建设前期工作。建成川黔铁路外迁工程，加快遵义至泸州铁路、城市轨道交通前期工作，力争开通遵义到贵阳和重庆的铁路公交专列。抓好务川至彭水、重庆至赤水、万盛至正安等高速公路前期工作，开工建设道真至务川、遵义至仁怀、金沙经仁怀至桐梓高速公路，力争开工道真至武隆高速公路，加快推进绥阳至正安、遵义至余庆、正安至习水、湄潭至石阡、崇遵扩容等高速公路建设，确保务川至德江、乐理至冷水坪高速公路建成通车。改造国省道260公里、县乡道100公里，建成撤并建制村硬化路3000公里以上。建成县级客运站4个，实现乡镇客运站全覆盖。加快桐梓河、芙蓉江等航运工程建设，开工建设余庆河等航运工程，确保构皮滩电站过船设施建成投用。加快水利能源通讯设施建设。开工建设观音大型水库，启动凤冈德隆等10座中小型水库建设，实现道真沙坝等10座水库下闸蓄水，治理病险水库5座以上，发展灌溉面积10万亩。加快页岩气开发利用，稳步推进风电项目，建成一批重点输变电工程，新改建10千伏及以下线路4488公里。持续推进“无限网络·满格遵义”“宽带乡村”“三网融合”（34）等信息基础设施建设，发展农村无线宽带用户5万户，持续抓好“多彩贵州”广电云户户用工程。

　　七、坚定不移推进改革开放创新。深化重点领域改革。推动投资结构改革，激活社会投资，实施市级以上重大工程项目852个，完成年度投资2223亿元，产业投资占比提高5个百分点，工业投资占比达22%以上。严格执行预算法和担保法，有效遏制隐性债务增量，稳妥处置隐性债务存量，切实防范化解政府性债务风险。深化金融市场化改革，调整优化存贷结构，降低企业融资成本，引导金融服务实体经济。深化国有企业改革，提升四大集团公司信用评级，增强融资能力，加快向市场化、实体化转型。深化投融资体制改革，创新政府资源资产优化配置，组建脱贫、旅游、康养、生态、工业发展基金和白酒、电子信息、军民融合、科技创新等子基金。深化城市管理综合执法、商事制度、“放管服”等改革，优化营商环境，新增市场主体6万户以上，民营经济比重达到57%。提高对外开放水平。加快推进国家内陆开放型经济试验区建设，全面深化渝遵战略合作，主动对接川渝，合力打造“成渝贵”内陆开放开发示范区,促进与川渝地区互联互通，加快融入“一带一路”和长江经济带发展。全力推动遵义综合保税区封关运行、加快发展。支持遵义高新区加强创新能力建设，力争升级为国家级高新区。积极推动遵义国家经济技术开发区、新蒲经济开发区扩区调位。加强遵义新舟机场国际航线培育，力争获批国家正式航空口岸。提升遵义（上海）产业园、上海漕河泾开发区遵义分区共建水平。加大外向型企业培育力度，打造仁怀白酒、正安茶叶、新蒲辣椒3个国家级出口质量安全示范区，新增外资企业10家以上，实际利用外资增长15%以上，新增外贸企业50家以上，进出口总额增长20%。增强创新驱动能力。像抓脱贫攻坚和教育优先发展一样抓科技创新，召开全市科技创新发展大会，大张旗鼓表彰有突出贡献的创新企业、创新团队、创新人才和创新成果。成功创建国家创新型城市。加大遵义院士工作中心建设力度。支持湄潭国家农业科技园区申建国家农业高新区。加大知识产权保护和成果转化力度，引导企业加大科研经费投入，新建或提升20个创新平台，培育省级以上科技型企业50家，申请发明专利1500件以上。深入实施人才强市战略，创新人才发展体制机制，全面推进“名城聚才”工程。

　　八、持之以恒加强生态文明建设。加强生态建设。全力建设国家生态文明试验区，严守生态保护红线、永久基本农田、城镇开发边界三条控制线。完成各类营造林90万亩，森林覆盖率达60%以上。持续推进山体公园、体育生态公园建设，建成35个乡镇森林公（游）园，建成1个以上省级森林特色小镇。全面推进山水林田湖草系统、生物多样性保护和地质灾害防治、石漠化治理，治理水土流失60平方公里、中小河流6条，力争创建省级生态文明建设示范县8个。推动绿色发展。加强绿色生产政策引导，推进农药、化肥零增长行动和生物防治病虫害，培育壮大节能环保、清洁能源产业，加快构建绿色低碳循环发展的经济体系。大力倡导绿色消费、绿色出行，深入开展节约型机关和绿色家庭、绿色学校、绿色社区创建行动。积极推进国家节水型城市创建。一般工业固废综合利用率提高到62%。强化环境治理。持续推进“双十”（35）工程和“治污治水·洁净家园”五年攻坚行动，全面加强大气、水、土壤、垃圾、固废等污染防治，坚决制止和惩处破坏生态环境行为。全面实施“河长制”，持续抓好乌江、赤水河、湘江河等流域生态环境保护与治理，不断提升污水处理设施建管运水平，确保打秋坪国控断面达到Ⅲ类水体。确保实现中心城区空气质量优良率94%、力争95%以上，城乡生活垃圾无害化处理率91%，医疗固废安全处置率保持100%，用水总量、用水效率、限制纳污“三条红线”低于省控目标。

　　九、千方百计保障和改善民生。坚持教育优先发展。推进校长职级制、教师薪酬、招生考试等基础教育领域综合改革，加快以“智慧校园”为载体的教育信息化建设，提升各级各类教育质量和教育对外开放水平，促进教育现代化。实施第三期学前教育三年行动计划、中心城区基础教育发展提升三年行动计划，扎实推进长征故事和中华优秀文化进校园。确保绥阳、正安县域内义务教育基本均衡发展通过国家评估。大力支持高等院校“双一流”（36）建设，继续推动申建遵义大学，支持遵义医学院更名遵义医科大学，支持遵义医药高等专科学校申建应用型本科学校，推进遵义职院、贵州航天职院建设发展。办好中国足球运动学院西南分院。大力促进就业创业。全面推进“技能兴遵”和全民创业行动计划，促进更高质量和更充分就业，新增城镇就业12.6万人，转移农村劳动力10.5万人，城镇登记失业率控制在4.2%以内。强化医疗卫生服务。深化医药卫生体制改革，推动多种形式“医联体”（37）建设，构建分级诊疗模式，不断提升“诊断、治疗、护理、管理、公卫”五种能力。完成基层医疗卫生服务能力三年提升计划，加快“百院大战”和重点科室建设。大力推进医疗卫生与养老服务融合发展。优化计生服务，完善全面两孩配套政策。繁荣发展文体事业。健全完善四级公共文化设施，创新开展“五大公共文化服务品牌”（38）。高标准办好省“十运会”、市第二届运动会，认真备战省第九届民运会，完善全民健身体育设施，推动群众体育、竞技体育和体育产业加快发展。办好第九届中国少年儿童合唱节。积极争取“中国第一方志之乡”“国家方志馆沙滩分馆”落户遵义。推动全民阅读。强化社会治理创新。深入开展“七五”普法，加强公共法律服务。加快“雪亮工程”（39）建设，持续推进严打整治斗争，大力整治群众反映强烈的突出治安问题，巩固全省最安全市州、西部最安全城市。强化矛盾纠纷排查化解，开展“信访积案清仓行动”，推动信访问题多元化解。构建“双重”预防机制（40），推进安全生产“六级六覆盖”（41），全面落实企业全员安全责任，抓好全国安全生产宣传教育“七进”（42）活动试点城市建设，落实安全隐患“五个一批”（43）整治要求，加大安全生产先进技术推广，坚决遏制重特大安全事故发生。与此同时，加快构建多层次的健康养老服务体系，抓好“双拥”创建、留守儿童和困境儿童关爱救助、防灾减灾救灾体系建设等工作。推动外事侨务、民族宗教、国防教育、人民防空、疾病防控、妇女儿童、档案管理、应急管理、老年人、残疾人、红十字会等事业再上新台阶。

　　我们将尽力而为、量力而行，全力办好十件民生实事。一是建成80个“全面改薄”项目，新（改、扩）建100所公办幼儿园，中心城区新（改、扩）建学校16所、新增学位1.4万个，建成市示范性青少年综合实践基地。二是全面完成市、县两级健康大数据信息平台建设。三是统筹开展贫困劳动力、城镇困难人员、农民工技能培训3万人，实现就业1.8万人。四是建成桃溪大道、人民路、南宁西路等9个中心城区路网改造项目。五是加快创建公交都市，新投放或更新城市公交车200辆，加密新蒲新区、南部新区公交线路。六是实施城镇棚户区改造7.9万套，改造农村危房3.2万户。探索实施进城落户农民工住房公积金制度。七是持续推进厕所革命，新建旅游公厕180所，完成所有学校旱厕改造。八是推进湘江系统整治，建成10座翻板坝和南部污水处理厂二期，实现干流截污管网全覆盖，完成干溪河、虾子河、蚂蚁河、忠庄河等支流水环境整治。九是继续推动大型文化、体育场馆免费或低收费开放，提升管理服务水平。十是完善“天网工程”，新增高清视频探头5000个，整合社会资源5000个。

　　十、驰而不息抓好政府自身建设。始终从严治政。深入贯彻新时代党的建设总要求，坚定理想信念宗旨不动摇，扎实开展“不忘初心、牢记使命”主题教育，严格遵守《党章》，严格执行《准则》和《条例》，进一步加强审计监督，拓展民生监督，切实做到政治坚定、持廉守正。坚持依法行政。完善政府立法机制，健全重大行政决策程序制度、合法性审查制度和终身责任追究制度及倒查机制，全面加强行政复议、行政调解、行政应诉、行政裁决，维护公平正义。自觉接受人大及其常委会的法律和工作监督、政协民主监督、新闻媒体和群众监督，高度重视民主党派、工商联和无党派人士意见，充分发挥工青妇等人民团体桥梁纽带作用，推进权力运行全流程、政务服务全过程公开，确保权力在阳光下运行。全面增强本领。开展学习型政府创建活动，用好新时代学习大讲堂，全面提升“八大本领”（44）。落实国家和省适应高质量发展的指标统计和绩效考评体系，大兴调查研究之风，大兴真抓实干之风，大兴勤俭节约之风，切实抢抓新机遇、积极应对新挑战、有效化解新矛盾、全力推动新发展。持续改进作风。充分发挥“头雁效应”，坚决贯彻中央和省委要求及市委十二项规定，突出惩治扶贫领域的腐败问题和不正之风。充分发扬“钉钉子”精神，加强正向激励，强化负面问责，完善容错机制，大力营造敢于担当、勇于负责的干事创业氛围。打铁必须自身硬。我们将深入查找和纠正“四风”突出问题新表现，重拳整治不作为、慢作为、乱作为等突出问题，为全市经济社会发展创造更加优质高效便捷的政务服务环境，让群众和企业办事像网购一样方便快捷。

　　各位代表，新时代催人奋进，新思想引领未来，新跨越全凭实干。让我们更加紧密地团结在以习近平同志为核心的党中央周围，大力弘扬“团结奋进、拼搏创新、苦干实干、后发赶超”的新时代贵州精神，咬定目标不放松，撸起袖子加油干，为开创百姓富、生态美的遵道行义·醉美遵义新未来而努力奋斗！
